# Supplementary material for: Mass spectrometry and Monte Carlo method mapping of nanoparticle ligand shell morphology
Source: Nat Commun. 2018 Oct 26;9:4478. doi: 10.1038/s41467-018-06939-y (PMC6203843; doi:10.1038/s41467-018-06939-y)
Supplement: Supplementary file 1 — Supplementary Information [file 41467_2018_6939_MOESM1_ESM.pdf]

SUPPLEMENTARY INFORMATION

Mass Spectrometry and Monte Carlo method mapping of nanoparticles ligand shell morphology

Z. Luo *et al.*

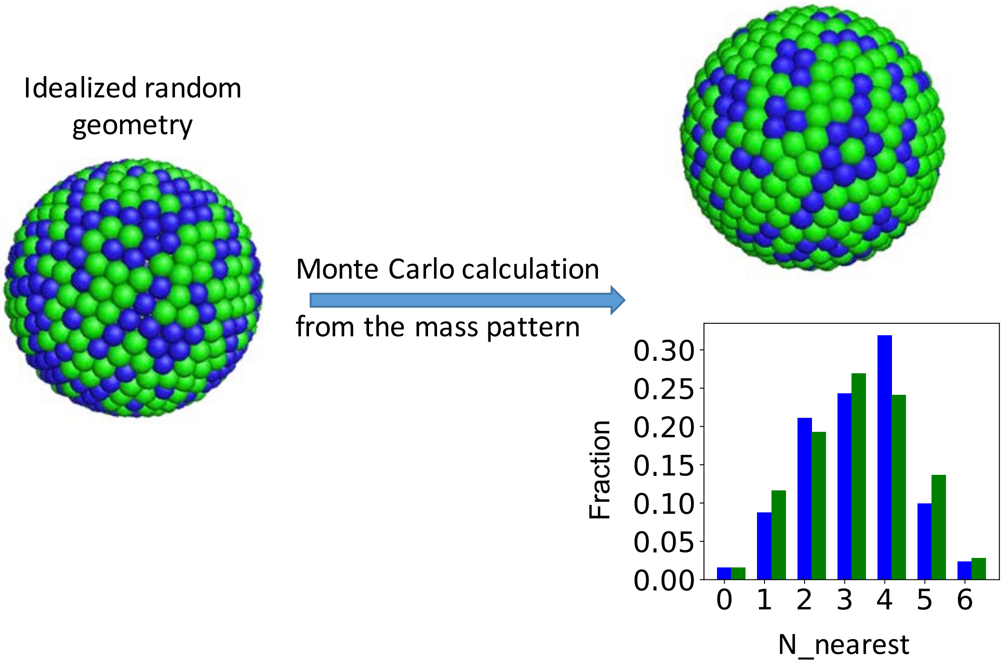

Supplementary Figure 1. Monte Carlo fitting of random LSM structures.

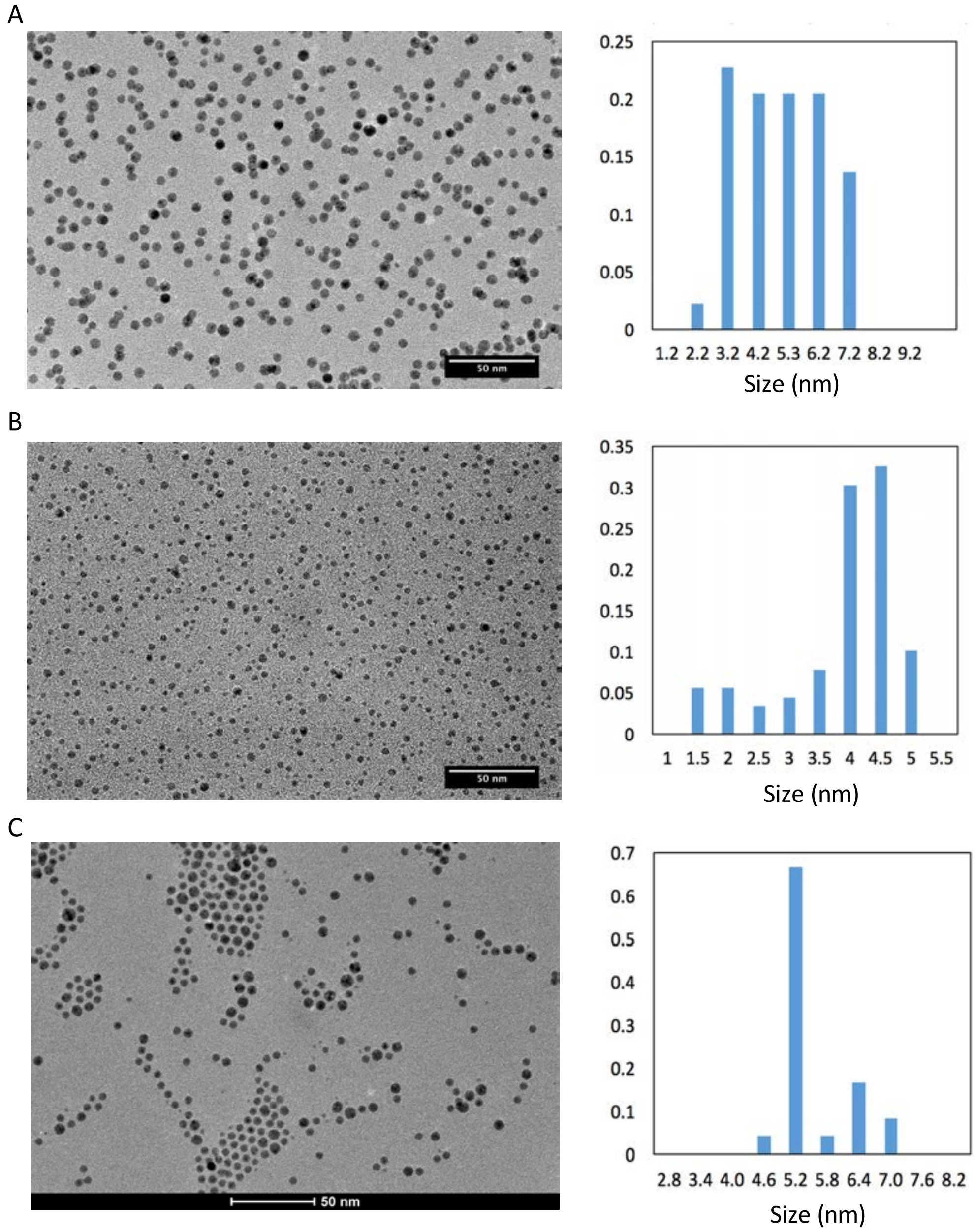

Supplementary Figure 2. TEM image of mixed ligand protected silver and gold nanoparticles. (A) Silver nanoparticle protected with PET and DDT. The average diameter is 5.8 ± 0.7 as shown in the histogram. (B) Gold nanoparticle protected with MUA and DDT. The average diameter is 4.1 ± 0.6 nm as shown in the histogram. (C) Silver nanoparticle protected with OT and DDT. The average diameter is 5.2 ± 0.7 nm as shown in the histogram.

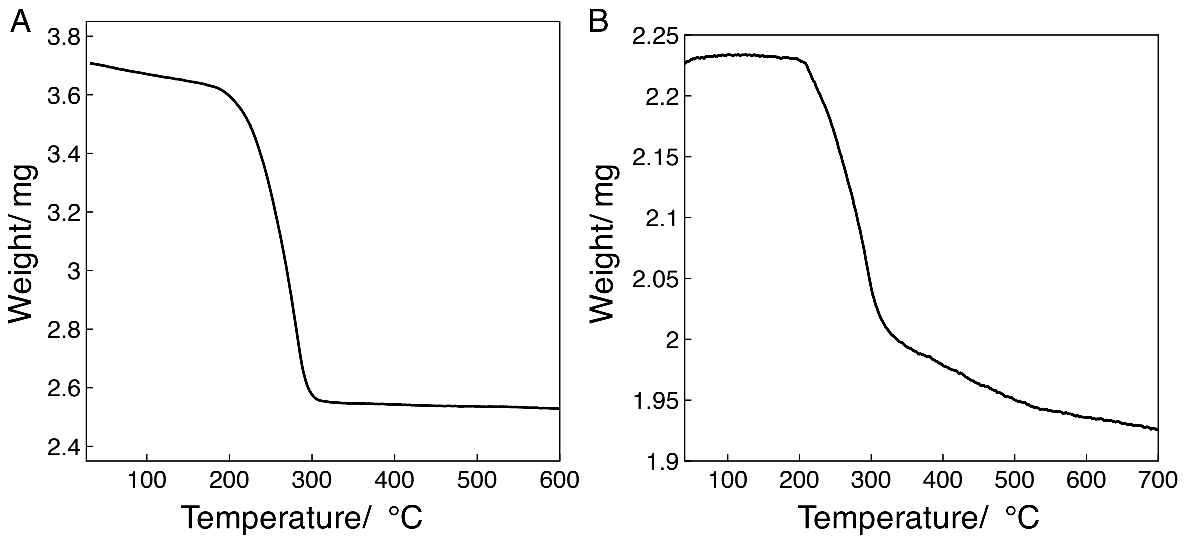

Supplementary Figure 3. TGA results of mixed ligand protected silver and gold nanoparticles. (A) TGA data of silver nanoparticle protected with PET and DDT. The percentage of ligands is 30% of the total nanoparticle weight. (B) TGA data of gold nanoparticle protected with MUA and DDT. The percentage of ligands is 13% of the total nanoparticle weight.

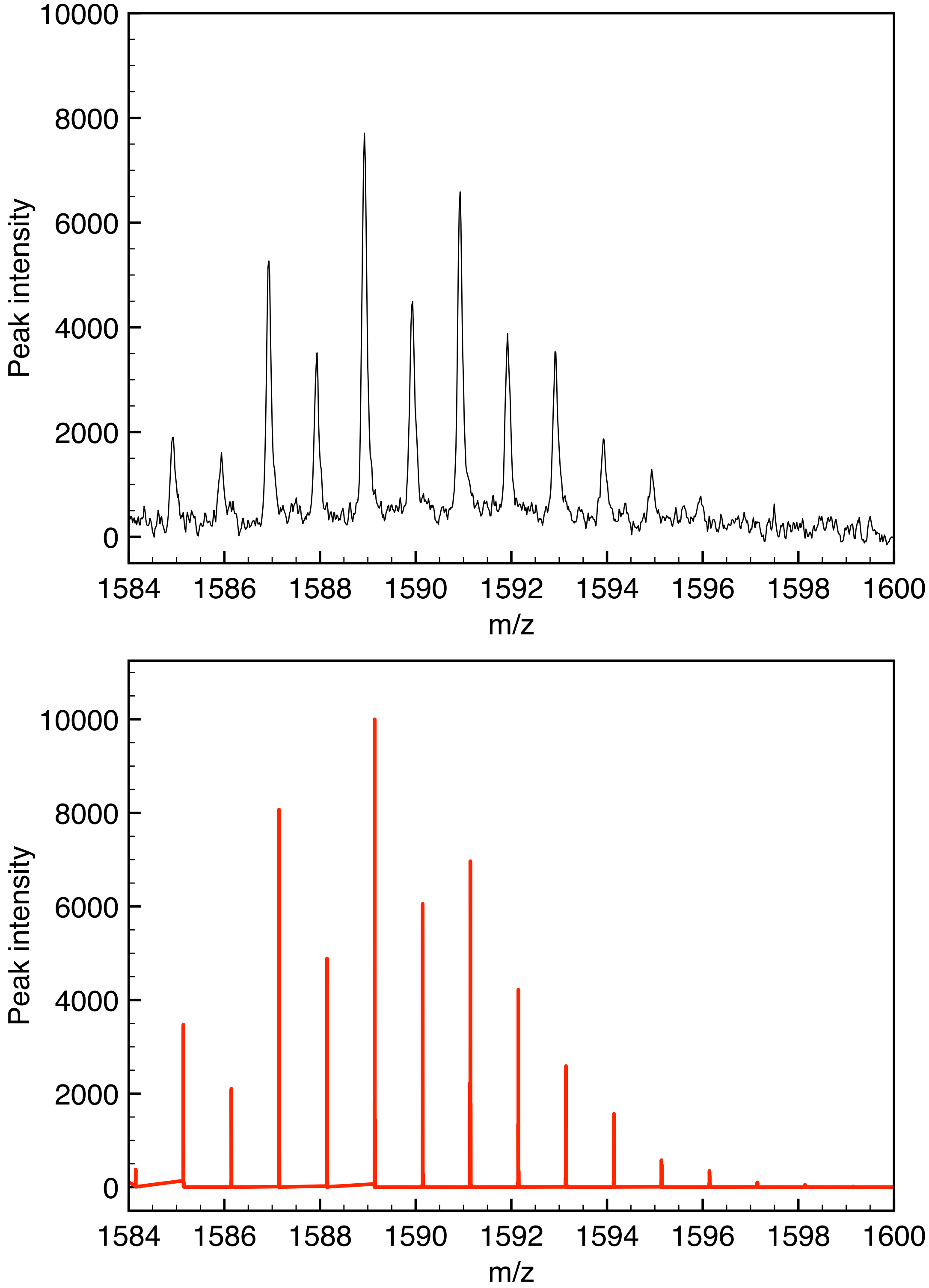

Supplementary Figure 4. Example of the comparison between experimental (up) and theoretical (bottom) isotope distribution profile of the fragments. The $Ag_6(DDT)_5(PET)_1$ is used as the example here. The mass accuracy is 50 ppm.

Supplementary Table 1. List of fragment masses and the regions for peak integration

Fragment	Average mass	Regions for integration
Ag5(DDT)4(PET)0	1344.941	1340-1351
Ag5(DDT)3(PET)1	1280.771	1276-1286
Ag5(DDT)2(PET)2	1216.601	1212-1221
Ag5(DDT)1(PET)3	1152.431	1148-1161
Ag5(DDT)0(PET)4	1088.261	1084-1094
Ag6(DDT)5(PET)0	1654.2092	1648-1661
Ag6(DDT)4(PET)1	1590.0392	1584-1600
Ag6(DDT)3(PET)2	1525.8692	1520-1531
Ag6(DDT)2(PET)3	1461.6992	1456-1468.2
Ag6(DDT)1(PET)4	1397.5292	1392-1404
Ag6(DDT)0(PET)5	1333.3592	1328-1339
Ag7(DDT)6(PET)0	1963.4774	1957-1971
Ag7(DDT)5(PET)1	1899.3074	1984-1906
Ag7(DDT)4(PET)2	1835.1374	1829-1842
Ag7(DDT)3(PET)3	1770.9674	1766-1777
Ag7(DDT)2(PET)4	1706.7974	1702-1712
Ag7(DDT)1(PET)5	1642.6274	1638-1648
Ag7(DDT)0(PET)6	1578.4574	1573-1583

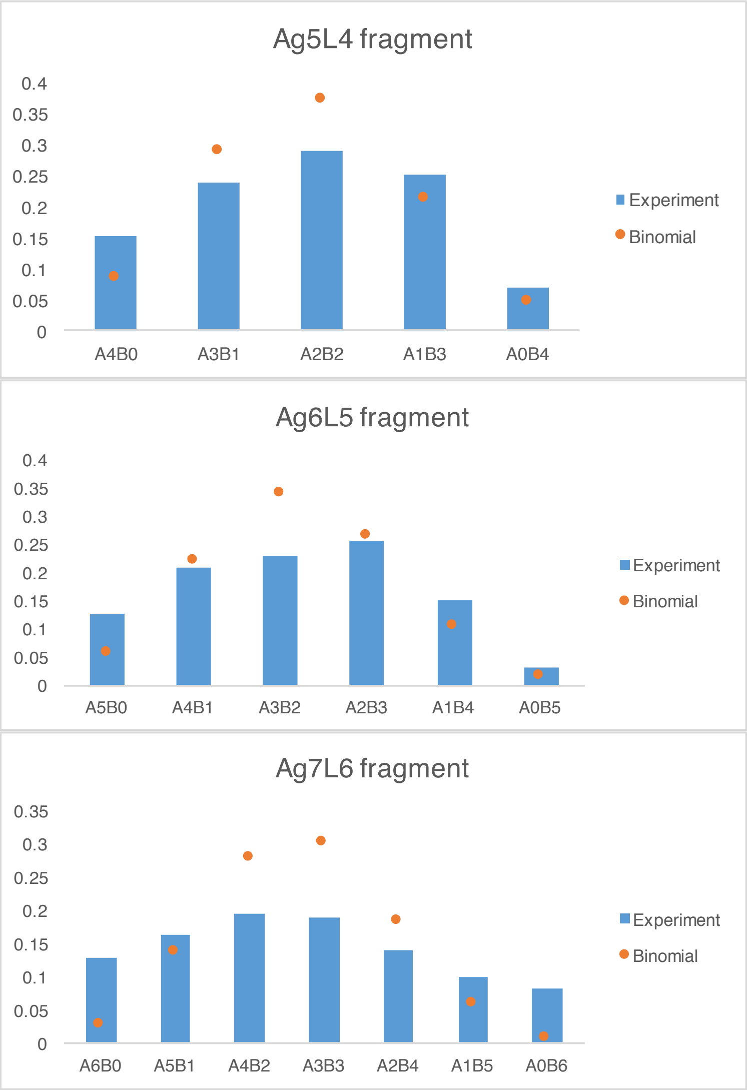

Supplementary Figure 5. Distribution of different fragments for PET-DDT silver nanoparticle. The blue charts represent the normalized integrated intensity of MALDI-TOF patterns. The orange dots stand for the binomial distribution at the same ligand ratio.

Supplementary Table 2. Ligand ratio and SSR value for the PET-DDT silver nanoparticle.

Fragments	Ligand ratio (PET: DDT)	SSR
Ag_5L_4	54% : 46%	0.016
Ag_6L_5	56%: 43%	0.019
Ag_7L_6	55%: 45%	0.039

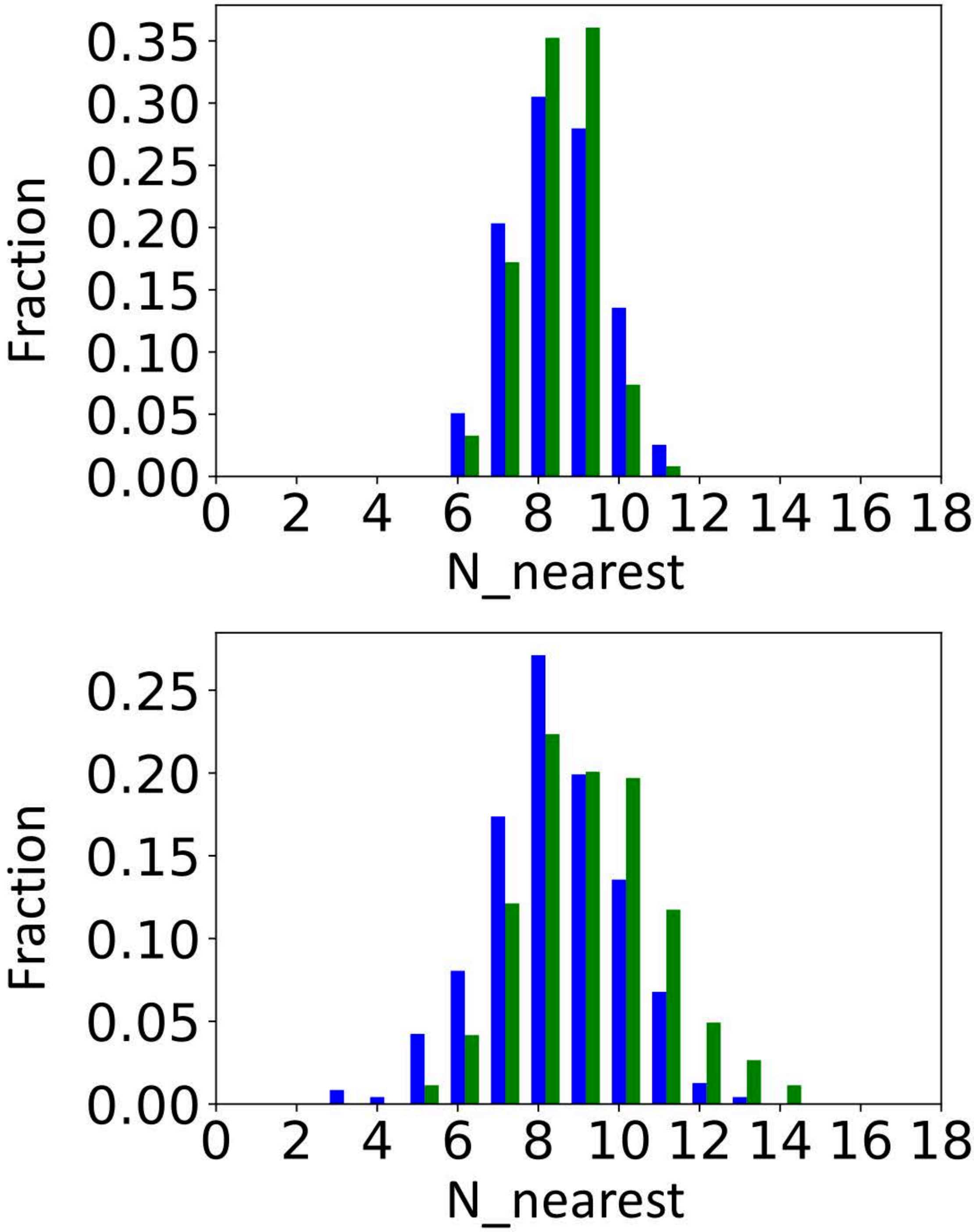

Supplementary Figure 6. Nearest neighbour distribution in the first two neighbouring shells (18 neighbours) of the idealized stripe-like (up) and PET-DDT AgNP (bottom) described in Figure 1F-G.

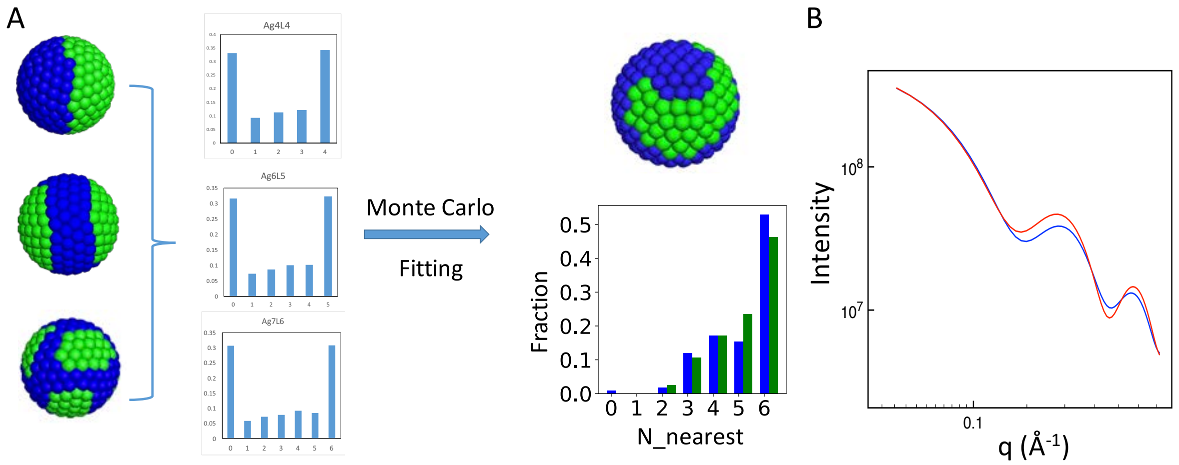

Supplementary Figure 7. Fitting the MALDI spectra of a mixture of different morphologies. (A) The theoretical MALDI spectra of the mixture were calculated by averaging the spectra of Janus and patchy nanoparticles with different patchy sizes. The Monte Carlo calculations after 10000 iteration steps gives a SSR value of 1.1 x 10^{-4}. The resulted model shows a morphology of interconnected patchy domains, which should be regarded as the averaged feature of the mixture. (B) Comparison of the theoretical SANS pattern for the mixture of three Patchy morphologies (red curve) and the model from Monte Carlo fitting (blue curve).

Supplementary Table 3. Comparison of the ratio of PET ligand in PET-DDT silver nanoparticles from NMR measurement and different fragments in MALDI-TOF MS.

Ratio from Ag_5L_4	Ratio from Ag_6L_5	Ratio from Ag_7L_6	NMR ratio
23%	23%	25%	23%
32%	34%	35%	35%
47%	46%	48%	42%
69%	67%	66%	65%
75%	74%	75%	71%
75%	75%	73%	70%
76%	76%	76%	74%
50%	50%	49%	50%
54%	54%	54%	53%
44%	45%	44%	46%

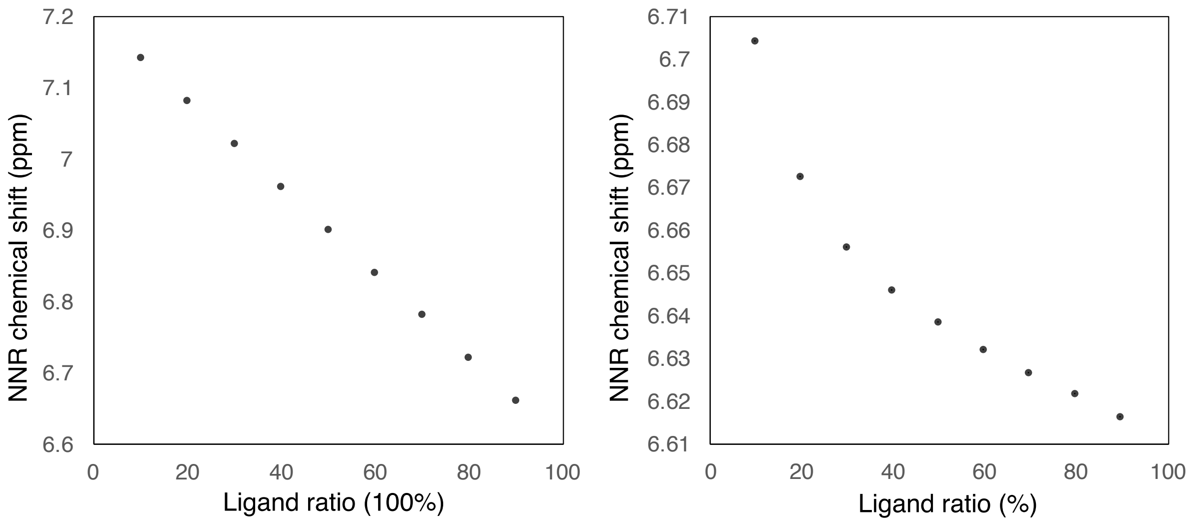

Supplementary Figure 8. Theoretical NMR chemical shift dependence on the ligand ratio for perfect random (left) and Janus (right) type of LSM. The chemical shift is a linear function of ligand ratio for random LSM while the dependence is close to $1/x$ for Janus LSM. The calculation is based on a 6 nm nanoparticle with 500 ligands.

Average PET% = 24%

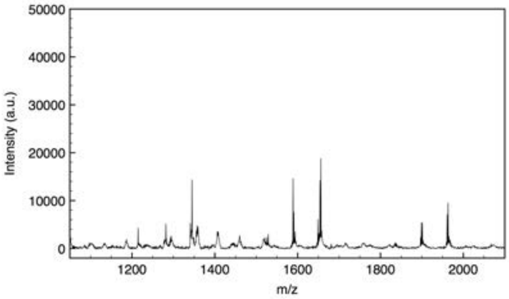

NMR PET% = 23%

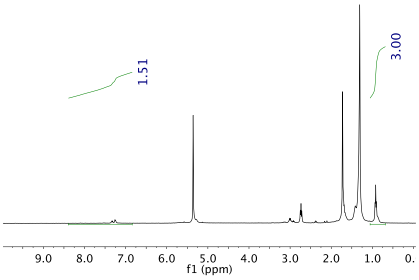

Average PET% = 34%

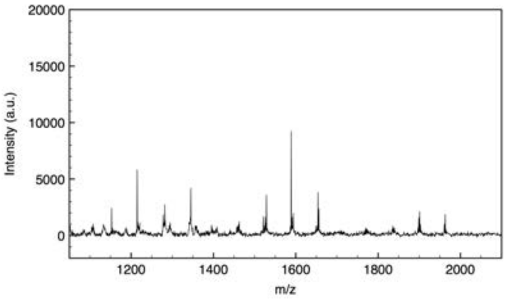

NMR PET% = 35%

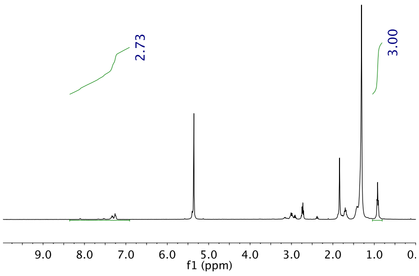

Average PET% = 42%

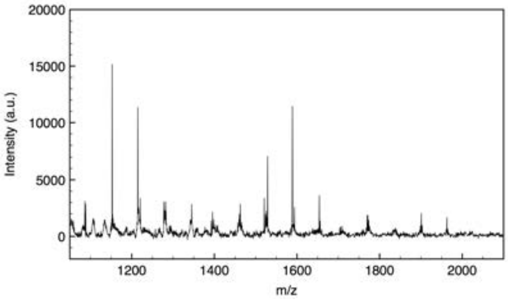

NMR PET% = 47%

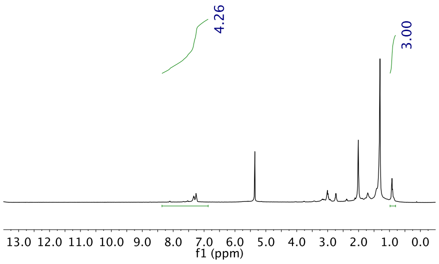

Average PET% = 50%

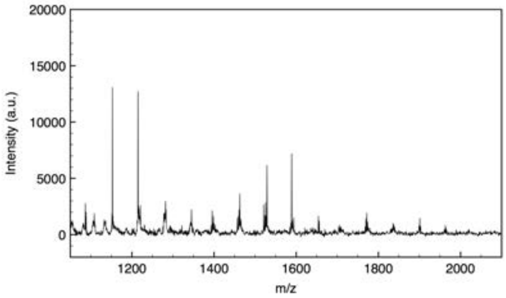

NMR PET% = 50%

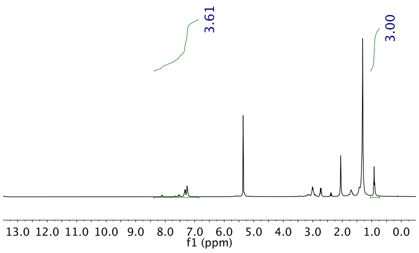

Average PET% = 54%

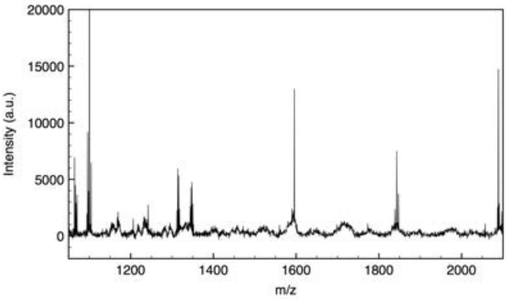

NMR PET% = 53%

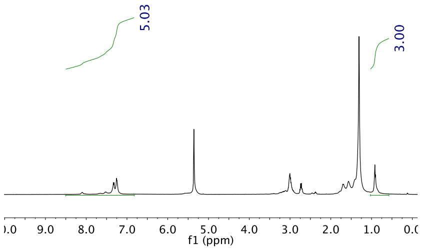

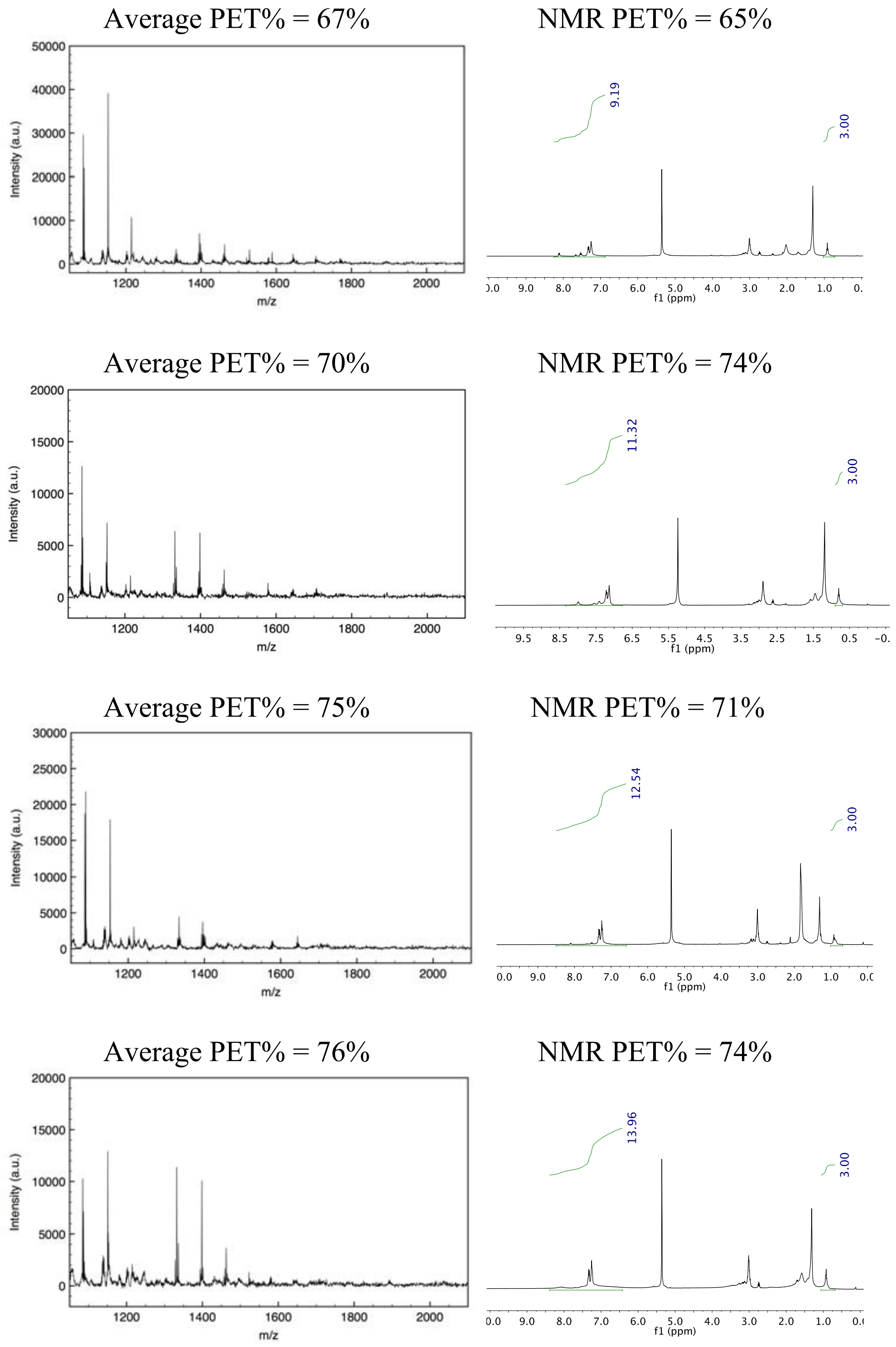

Supplementary Figure 9. MALDI-TOF (left) and NMR spectra (right) of the PET-DDT AgNPs with varying ligand ratios. The ligand ratio calculated from MALDI and NMR are labelled on top of each spectra.

Supplementary Discussion:

Tests on various samples

We continue to use this method to the analysis of nanoparticles coated with different ligand mixtures. Silver nanoparticles protected by a mixture of butanethiol-DDT, hexanethiol-DDT and octanethiol-DDT ligands were studied. These samples were chosen as they have been previously studied by MALDI-TOF and compared with computer simulations[1,2]. The three models and nearest neighbor distributions are shown in Supplementary Figure 10A-C and results indicate that all these three nanoparticles have patchy type structures with varying patch sizes.

Finally, the same analysis procedure was performed on gold NPs to further demonstrate the versatility of this method. Gold NP with a core diameter of 4.1 ± 0.6 nm and protected with 11-mercaptoundecanoic acid (MUA) and DDT ligands was synthesized as described in Method section, Supplementary Figure 10E. The TEM (Supplementary Figure 1) and TGA (Supplementary Figure 2) measurements indicate that 240 ligand molecules exist on one nanoparticle surface. The three types of fragments, Supplementary Figure 10D, give a ligand ratio of MUA: DDT= 74%: 26%. As shown in Supplementary Figure 11 and Table S4 the variation of ligand ratios among different fragments is less than 5%. The model generated from Monte Carlo calculation give a SSR value of 9×10^{-4}, as shown in Supplementary Figure 10F. One can see from the model that DDT ligand forms some dimers and trimers on the NPs surfaces, as also indicated by the nearest neighbor profile.

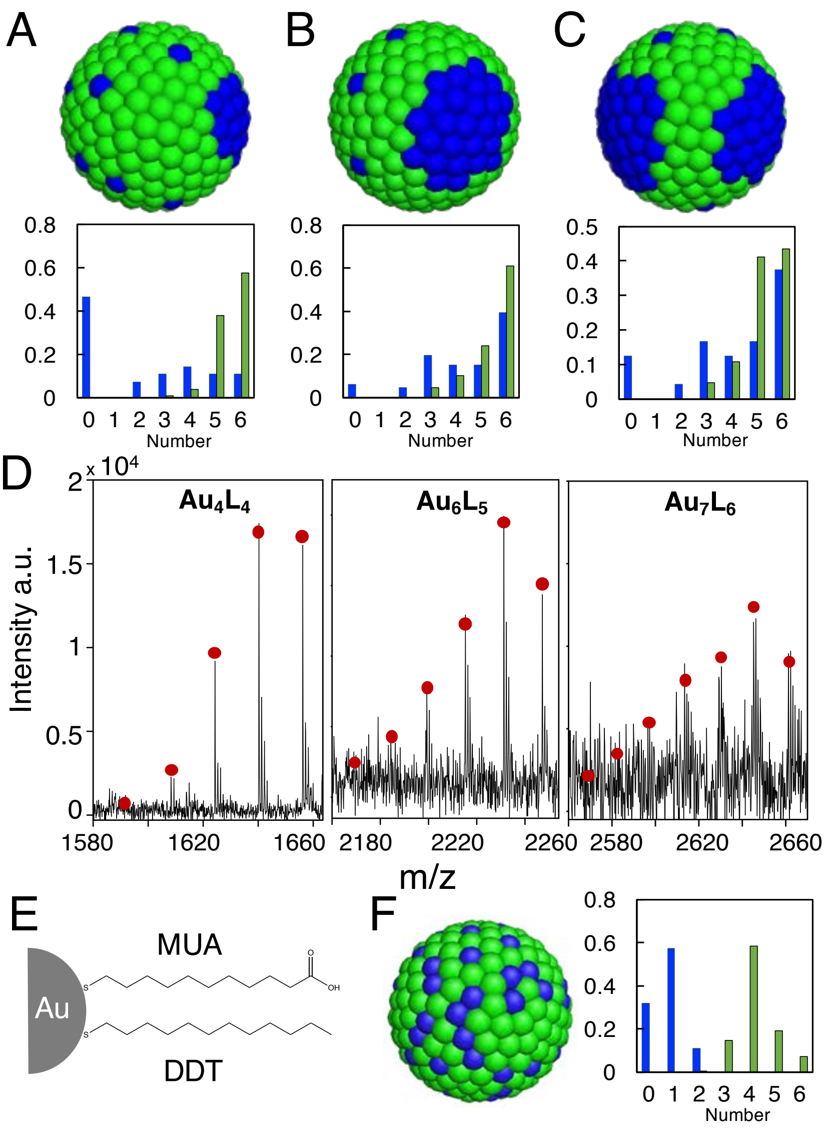

Supplementary Figure 10. Modeling the MALDI-TOF data on various nanoparticles. Silver nanoparticles protected with (A) butanethiol- DDT, (B) hexanethiol-DDT and (C) octanethiol-DDT ligands are modelled together with their nearest neighbor profiles. The x axes of the profiles stand for the number of nearest neighbors of ligand A with the same identity of A. The blue and green columns correspond to beads with blue and green colors respectively. (D) MALDI-TOF MS data of gold NPs protected by MUA and DDT ligands. Red dots mark the intensity for the fragmentation of calculated model. (E) Schematics of the chemical structure of the ligands. (F) Model and the corresponding nearest neighbor distribution from the Monte Carlo fitting of the MALDI spectra.

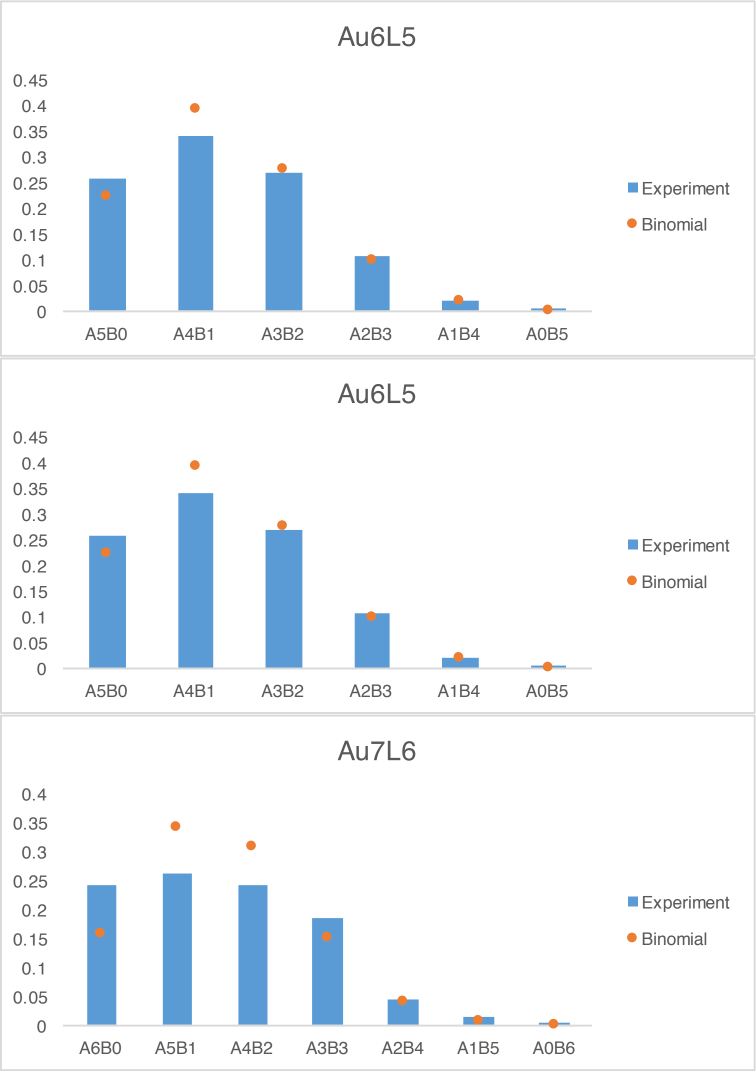

Supplementary Figure 11. Distribution of different fragments for MUA-DDT gold nanoparticle. The blue charts represent the normalized integrated intensity of MALDI-TOF patterns. The orange dots stand for the binomial distribution at the same ligand ratio.

Supplementary Table 4 Ligand ratio and SSR value for the MUA-DDT silver nanoparticle.

Fragments	Ligand ratio (MUA: DDT)	SSR
Au4L4	74% : 26%	0.004
Au6L5	74%: 26%	0.004
Au7L6	73%: 27%	0.010

Supplementary References

1. Farrell, Z. *et al.* Development of Experiment and Theory to Detect and Predict Ligand Phase Separation on Silver Nanoparticles. *Angew. Chemie* **127,** 6579–6582 (2015).
2. Merz, S. N. *et al.* Theoretical and Experimental Investigation of Microphase Separation in Mixed Thiol Monolayers on Silver Nanoparticles. *ACS Nano* **10,** 9871–9878 (2016).
